# Yolk sac primary tumor of mediastino: a rare case in a young adult

**DOI:** 10.1590/S1679-45082017RC4008

**Published:** 2017

**Authors:** Lorena Luryann Cartaxo da Silva, Fernanda Sasaki Vergilio, Diva Carvalho Collarile Yamaguti, Isabela Azevedo Nicodemos da Cruz, Joana Angrisani Granato Queen

**Affiliations:** 1 Instituto de Assistência Médica ao Servidor Público Estadual, Hospital do Servidor Público Estadual “Francisco Morato de Oliveira”, São Paulo, SP, Brazil.

**Keywords:** Neoplasms, germ cell and embryonal, Endodermal sinus tumor/drug therapy, Mediastinal neoplasms, Neoadjuvant therapy, Case reports, Neoplasias embrionárias de células germinativas, Tumor do seio endodérmico/tratamento farmacológico, Neoplasias do mediastino, Terapia neoadjuvante, Relatos de casos

## Abstract

Germ cell tumors are rare neoplasms that mostly occur in the gonads, although they can also affect other body sites, especially the anterior mediastinum (50 to 70% of all extragonadal germ cell tumors). We report a case of a primary mediastinal yolk sac tumor, a rare and aggressive germ cell tumors subtype. This was a 38-year-old man who was admitted to *Hospital do Servidor Público Estadual “Francisco Morato de Oliveira”,* complaining about dyspnea and dry cough for 1 year. The computed tomography scan of his chest revealed a large mass in the anterior mediastinum with heterogeneous enhancement to the contrast associated with pleural effusion. There were also high serum levels of alpha-fetoprotein. After neoadjuvant chemotherapy, the patient underwent surgical resection of the mass, followed by pathological examination, which confirmed a primary mediastinal yolk sac tumor, a nonseminomatous subtype of germ cell tumors. Primary mediastinal yolk sac tumors have poor prognosis, despite advances in therapy with surgical resection and cisplatin-based chemotherapy. This poor prognosis is due to the degree of invasion and unresectability in most patients by the time of the diagnosis.

## RESUMO

Os tumores de células germinativas são neoplasias raras que acometem mais frequentemente as gônadas, embora possam também ocorrer em outras localizações do corpo, destacando-se o mediastino anterior (50 a 70% de todos os tumores de células germinativas extragonadais). No presente artigo, relatamos um caso de tumor de saco vitelínico mediastinal primário, de subtipo raro e agressivo de tumor de células germinativas. Tratava-se de um homem, 38 anos, admitido no Hospital do Servidor Público Estadual “Francisco Morato de Oliveira”, com quadro de dispneia e tosse seca há 1 ano. Na investigação clínica, foi solicitada tomografia computadorizada de tórax, que mostrou volumosa massa no mediastino anterior com realce heterogêneo ao meio de contraste associada a derrame pleural. Havia ainda aumento dos níveis séricos da alfafetoproteína. Após quimioterapia neoadjuvante pré-operatória, o paciente foi submetido à ressecção cirúrgica, seguida de estudo anatomopatológico da peça, no qual demonstrou tratar-se de um tumor de saco vitelínico primário do mediastino. Os tumores de saco vitelínicos primários do mediastino têm prognóstico reservado, apesar do avanço na terapêutica com a ressecção cirúrgica e a quimioterapia à base de cisplatina. Isto se deve ao grau de invasão e de irressecabilidade na maioria dos pacientes no momento do diagnóstico.

## INTRODUCTION

Germ cell tumors (GCT) are rare neoplasms that often affect gonads, although they can also occur in other body sites, mainly in structures of median line, such as pineal gland, retroperitoneum, anterior mediastinum and sacrococcygeal region.^1^

Among tumors of extragonadal origin, we highlight the primary mediastinal GCT that correspond 10% to 20% of all mediastinal cancers. These tumors are classified into two main categories: seminomas and non-seminomas, and non-seminomas sub-classified in yolk sac, choriocarcinoma, embrionary carcinoma and mixed germ cell tumors.^2^

Concerning subtype mentioned, we highlight primary mediastinal yolk sac, also called endodermal sinus tumor, first described by Teilmann et al.^3^ These tumors are aggressive and highly malignant, which can be explained by fast grown of germ cells, and normally unresectable at diagnosis. In this paper, we report a case of primary mediastinal yolk sac, rare subtype and highly malignant of GCT.

## CASE REPORT

A 38-year-old man admitted at *Hospital do Servidor Público Estadual “Francisco Morato de Oliveira”,* complaining about dyspnea to efforts and dry cough for 1 year. Upon physical examination, a reduction of vesicle murmur in left hemithorax was observed. Systemic arterial hypertension and hyperuricemia were the preexisting conditions.

**Figure 1:**
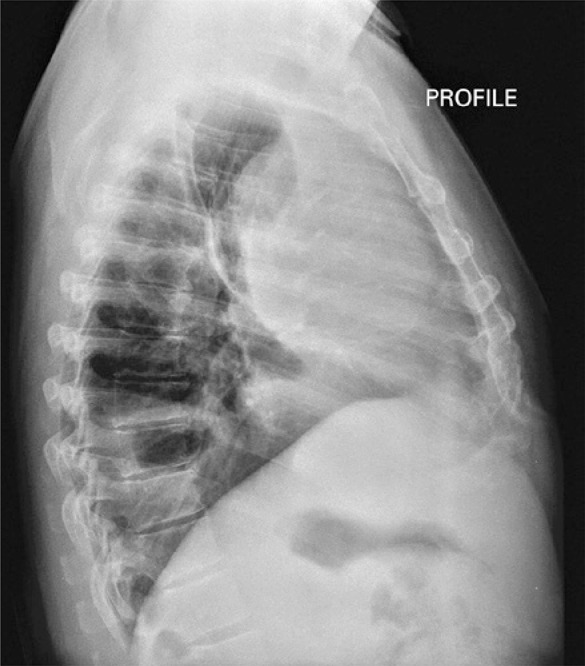
Profile of the thorax radiography showing homogenous densification located in anterior mediastium suggesting round contouring large mass

**Figure 2:**
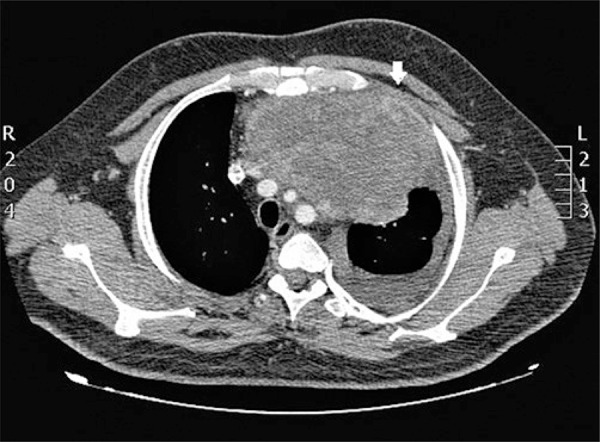
Thorax tomography scan with contrast media, axial slice, showing voluminous expansive formation in anterior mediastinum without clear cleavage plan with supra-aortic branches and superior cava vein, and erasure of extrapleural fat (arrow), which suggested invasion

**Figure 3:**
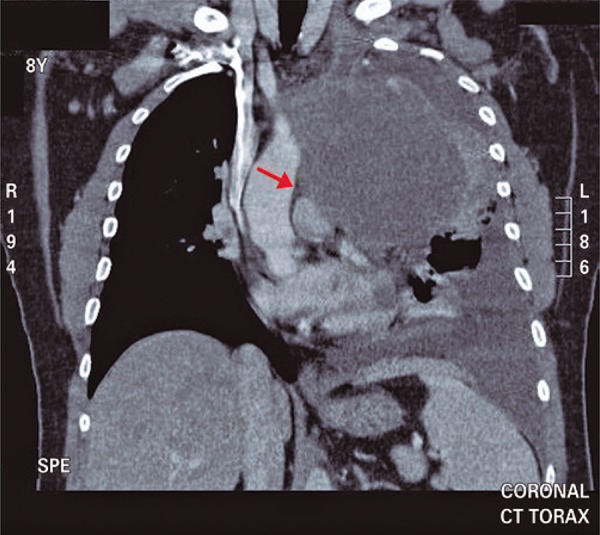
Thorax tomography with contrast media showing the contact of mediastium mass with aorta a pulmonary trunk (arrow). A stroke to left is seen and also compressive atelectasis of pulmonary parenchyma adjacent to injury

The thorax radiography upon admission showed homogeneus densification of anterior mediastinum, suggesting round contouring mass and pleural reaction of septate aspect of left hemithorax (Figure 1). The following exam, thorax tomography, showed anterior mediastinal mass with post-contrast heterogenous highlight and predominantly peripheral, measuring 9.1x14.6x11.6cm, therefore, showing broad contact with aortic arch, pulmonary artery trunk, sternum and thoracic wall (Figure 2). Still, there was stroke to left and laminar to right, as well as, pericardic effusion (Figure 3). We did not observe abnormalities in computed tomography scans of abdomen and skull.

The patient was followed-up in the thoracic surgery unit, and exams were requested to clarify the clinical picture. Exams showed elevated and rising alpha-fetoprotein (greater than 170mg/mL), without elevation of other serum tumor markers. Ultrasonography of testicle sac did not show changes.

Neoadjuvant chemotherapy was initiated with bleomycin, etoposide and cisplatin, there was an increase of serum alpha-fetoprotein level. We decided to change scheme for combination of paclitaxel, ifosfamide, and cisplatin (TIP) in case of clinical response associated with reduction tumor marker.

Patient underwent thoracic surgery for resection of mediastinal mass. After the surgery, he evolved with septic shock with pulmonary focus, and he was transferred to intensive care unit. After stabilize, the patient underwent new exams to disease restaging, and he was referred to clinical oncology to continuing the chemotherapy.

### Anatomopathological findings

The macroscopy observed the mediastinal mass with extensive necrotic-hemorrhagic areas infiltrating the pulmonary parenchyma by contiguity with focal vascular permeation. The resected fatty mediastinal mass as well as pericardium and surgical margins free of cancer.

**Figure 4:**
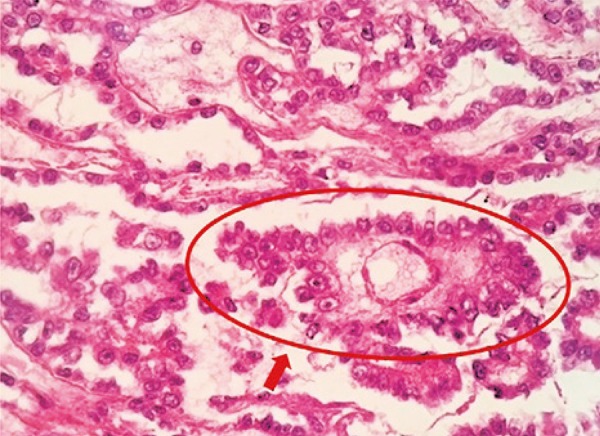
Histopathology of yolk sac tumor (hematoxylin and eosin staining, 20x). Schiller-Duval body (arrow)

The histopathology showed reticular area with glandular-like structures and prominent nucleus in direction to the lumen. There was surrounded vessels by fibrous tissue located in cystic space coated by neoplastic cells radially lined forming a Schiller-Duval body, pathognomonic findings of yolk sac tumor, therefore, confirming the diagnosis of GCT of primary yolk sac tumor of mediastinum (Figure 4).

## DISCUSSION

Extragonadal primary GCT account for 1% to 5% of all GCT, and mediastinum is the most common site, constituting 50 to 70% of all extragonadal GCT.^4^ These tumor probably derivate from fails in migration of primitive germ cells that migrate through urogenital crest during embryogenesis to develop regular function and hematological and immunological transport information.

We report a case of young man with yolk sac tumor, a type of non-seminomas GCT.

This disease affect mainly young men and it occurs as anterior mediastinal masses (most commonly in extragonadal origin), locally invasive, that trigger early metastasis by lymphatic and hematogenous metastasis, observed in approximately 25% of patients upon diagnosis.^5,6^ In our report, the patient did not present lymph nodal affection nor secondary dissemination.

Symptoms depend on size of the injury. Bokemeyer et al*.*, reported in a study that most common symptom in patients with GCT are dyspnea (25%), thoracic pain (23%), fever (13%) night sweats or weight loss (11%). Fatigue, hemoptysis and superior vena cava syndrome were seen in less than 10% of patients with mediastinal GCT.^1,7^

Some clinical conditions are associated with yolk sac tumor, according to literature, such as Klinefelter syndrome (around 20% of cases), precocity of pubertal development and hematological neoplasia, such as leukemia and myelodysplastic syndrome.^2,8,9^

In yolk sac tumor, alpha-fetoprotein is elevated, and in some cases carcinoembrionary antigen can also be elevated.^8^Alpha-fetoprotein serum levels are useful for diagnosis, and, mainly, in the follow-up of this tumor, therefore showing if there is or not a response to treatment.

Standard treatment for mediastinal primary non-seminomas GCT is combination of surgical resection with neoadjuvant systemic chemotherapy with bleomycin, etoposide and cisplatin.^2,5^However, our patient did not respond well to treatment, and we need to submit him to TIP. This fact corroborated with other studies that highlight prognosis reserved to these tumors with failure on rescue therapy, as well as high indexes of local recurrence compare with non- seminomas GCT of other local.

## CONCLUSION

Primary mediastinal yolk sac tumor is an extremely rare tumor, diagnosed using image finding, increase of alpha-fetoprotein serum levels and histopathological evaluation of the injury. These local invasive tumors present a reserved prognosis and high recurrence rates, although there was an increase of patients’ survival after implement cisplatin-based chemotherapy. Therefore, patients with this disease need early diagnosis and specialized and interdisciplinary management.
